# Comparative analysis of stalked and acorn barnacle adhesive proteomes

**DOI:** 10.1098/rsob.210142

**Published:** 2021-08-18

**Authors:** Janna N. Schultzhaus, William Judson Hervey, Chris R. Taitt, Chris R. So, Dagmar H. Leary, Kathryn J. Wahl, Christopher M. Spillmann

**Affiliations:** ^1^ Center for Bio/Molecular Science and Engineering, Naval Research Laboratory, Washington, DC, USA; ^2^ Chemistry Division, Naval Research Laboratory, Washington, DC, USA

**Keywords:** Pedunculata, Sessilia, *Pollicipes pollicipes*, *Amphibalanus amphitrite*, cement proteins, mass spectrometry

## Abstract

Barnacles interest the scientific community for multiple reasons: their unique evolutionary trajectory, vast diversity and economic impact—as a harvested food source and also as one of the most prolific macroscopic hard biofouling organisms. A common, yet novel, trait among barnacles is adhesion, which has enabled a sessile adult existence and global colonization of the oceans. Barnacle adhesive is primarily composed of proteins, but knowledge of how the adhesive proteome varies across the tree of life is unknown due to a lack of genomic information. Here, we supplement previous mass spectrometry analyses of barnacle adhesive with recently sequenced genomes to compare the adhesive proteomes of *Pollicipes pollicipes* (Pedunculata) and *Amphibalanus amphitrite* (Sessilia). Although both species contain the same broad protein categories, we detail differences that exist between these species. The barnacle-unique cement proteins show the greatest difference between species, although these differences are diminished when amino acid composition and glycosylation potential are considered. By performing an in-depth comparison of the adhesive proteomes of these distantly related barnacle species, we show their similarities and provide a roadmap for future studies examining sequence-specific differences to identify the proteins responsible for functional differences across the barnacle tree of life.

## Graphical abstract

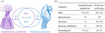

## 1. Introduction

Barnacles are sessile crustaceans that aggressively colonize marine surfaces, often crowding out other micro- and macrofouling organisms. Barnacle species exhibit a remarkable breadth of diversification both physically, exhibiting either a stalked or acorn form, as well as in terms of the environmental conditions and the types of surfaces on which their larval forms settle and grow. Some species are generalists, opportunistically growing on any suitable surface they encounter in the marine environment, whereas others are specialists, growing only on specific hosts (e.g. sea turtles [1], whales [2] and coral [3]) or in narrowly defined environmental ranges. This diversity, combined with globalization of the shipping industry, has enabled the spread of barnacles throughout the world's oceans, yet their success is also due to a common, novel trait: adhesion. In general, barnacles produce a highly proteinaceous adhesive to permanently cement themselves to the substrate [4]. Mechanical aspects play a role in adhesion, particularly in acorn barnacles where shell geometry [2] and base plate mechanical properties [5,6] are major contributors. While the exact mechanisms of how proteins contribute to barnacle adhesion are unknown, many have been proposed [7–9], including specific protein–protein interactions [10–13], repetitive sequence motifs [14] that drive nanofibril formation [15–19], evolution of novel adhesive functions from ancestral wound healing processes [20] and oxidative modification of proteins [21]. All evidence points to barnacle adhesion being distinct from other well-studied marine adhesive processes which often rely on serine phosphorylation or DOPA [22,23].

Researchers have traditionally divided the superorder Thoracica into the Pedunculata (stalked) and Sessilia (acorn) orders, although the Pedunculata are not a monophyletic group [24]. While many Thoracican species have been studied for a variety of reasons, *Pollicipes pollicipes* and *Amphibalanus amphitrite* have emerged as two model organisms for stalked and acorn barnacles, respectively. *P. pollicipes* belong to one of the most basal Thoracican families, the Pollicipedidae, while *A. amphitrite* belong to one of the most derived families, the Balanidae [24]. Both species are of interest commercially, but for different reasons. *P. pollicipes* is a delicacy in parts of Europe, garnering interest in aquaculture and population management strategies to improve production [25,26]. As a pervasive species with a cosmopolitan distribution, *A. amphitrite* has become a model fouling organism [27] due to its strong adhesion and calcareous shells [28]. With this backdrop, an increased understanding of adhesive composition and function is useful for both improving husbandry of barnacle food stocks as well as controlling barnacle biofouling.

The composition of the adhesive and how it varies across the barnacle evolutionary tree [29] is incompletely understood due, in large part, to a lack of genetic data. Early studies sequenced a small number of the most abundant proteins in the partially solubilized adhesive of acorn barnacles [10,23,30] and these proteins were named after the species of origin and protein molecular weight (i.e. Mrcp100 k for *Megabalanus rosa* cement protein 100 kDa). More recent studies have taken this a step deeper by developing transcriptomic-based protein databases [31,32] for proteomic assessment of both stalked [33,34] and acorn barnacles [14,21,35]. Concomitantly, there has been a dramatic increase in the number and types of proteins identified in barnacle adhesive, highlighting the fact that a number of complex biological processes in addition to adhesion occur at the barnacle base, contributing to growth and cuticle formation and breakdown. After decades of access to limited genomic information the genomes of four barnacle species have now been assembled (*P. pollicipes* (RefSeq GCF_011947565.2); *A. amphitrite* [36]; *Balanus improvisus* [37]; *Semibalanus balanoides* [38]), offering new opportunities to understand the coordinated processes which culminate in expansion of the adhesion interface to the substrate.

Although differences between the adhesives of stalked and acorn barnacles have been noted [39], a detailed comparison of the proteins present in the adhesive proteomes has not been performed. Here, raw mass spectrometry (MS) proteomic data from previous studies of the adhesive of *P. pollicipes* [33] and *A. amphitrite* [35] are reanalysed using the recently available genomic information, and the resulting adhesive proteomes are compared with focus given to identifying homologues between the species. Identified proteins are divided into functional categories and their sequences and structural properties are assessed. Although several major differences are noted between the adhesive proteomes, these two distantly related species show significant overlap with similar types of proteins, suggesting that the evolution of barnacle adhesive may have been constrained during species radiation. This first step in comparing the proteomes of the adhesive material from these divergent species provides a pathway for biomolecular studies of individual proteins that vary between species to promote an understanding of how these adhesives have developed.

## 2. Methods

### 2.1. Mass spectrometry analysis of *A. amphitrite* adhesive

Collection and sample processing of adhesive samples from *A. amphitrite* are previously described [35]. The hydrated, thickened barnacle adhesive was collected from the base of individuals dislodged from silicone panels, and samples were treated with three solvents (hexafluoroisopropanol [HFIP], urea or methanol) and extracted using pressure cycling technology (*n* = 3 for each solvent). These samples were analysed using a Triple TOF 5600+ mass spectrometer (AB Sciex, Foster City, CA, USA) [35]. Here, the samples are reanalysed with an Orbitrap Fusion Lumos Tribrid (Thermo Scientific, Waltham, MA, USA) mass spectrometer (electronic supplementary material, figure S1*a*).

Liquid chromatography tandem MS (LC-MS/MS) was performed with a U3000 HPLC system (Thermo Scientific, Waltham, MA, USA) coupled to an Orbitrap Fusion Lumos Tribrid mass spectrometer. The U3000 system was configured for one-dimensional nanoflow separations with on-line desalting. The autosampler injected and concentrated peptide samples onto a trap column (PepMap 100, C18, 300 µm ID × 5 mm, 5 µm, 100 Å) with the loading pump at a flow of 2% solvent B (0.1% formic acid in acetonitrile) and 98% solvent A (0.1% formic acid in water) at 5 µl min^−1^. After desalting for 3 min, the flow was diverted in-line at 300 nl min^−1^ for separation across a reverse phase analytical column (Acclaim PepMap RSLC, 75 µm ID × 150 mm, C18, 2 µm, 100 Å) for a total duration of 120 min. A two-step gradient of increasing solvent B (18% over first 80 min, followed by an increase of 60% over 15 min) was used to elute peptides off the column for MS analysis. Nanoelectrospray voltage was applied via the ion source with a stainless-steel emitter tip.

MS data were recorded on the Orbitrap Fusion Lumos Tribrid in data dependent mode with dynamic exclusion enabled. Each sample was analysed three separate times to collect data with the Orbitrap (OT) and the IonTrap (IT) analysers after high-energy collisional (HCD) or collision-induced (CID) dissociation (HCD/IT, CID/IT and HCD/OT methods). XCalibur (v. 4.2) was used to acquire profile measurements with the following settings: 20 scans per cycle were performed with a survey scan range of 400–1600 Da using the Orbitrap detector (resolution 120 K) for MS1 scans; the maximum injection time was set to 100 ms and the automatic gain control (AGC) target was 1.06; the most intense ions with charges of 2–5 were fragmented using 30% HCD or 35% CID for 10 ms; ions were excluded for 15 s from subsequent MS/MS submission after one time with a +50 ppm error tolerance; fragment ions were measured in the IonTrap (rapid rate, 35 ms maximum injection, AGC target = 10 000) or the Orbitrap detector (50 000 resolution, 86 ms maximum injection, AGC target = 50 000).

### 2.2. Mass spectrometry analysis of *P. pollicipes* adhesive

Collection and sample processing of adhesive samples from *P. pollicipes* [33] are already described. Briefly, the collected *P. pollicipes* adhesive proteins were extracted following an adapted single-pot solid-phase-enhanced sample preparation protocol. The digested peptides were analysed using a U3000 HPLC system coupled to an Orbitrap Fusion Lumos Tribrid mass spectrometer. Ion fragmentation and measurement were carried out with HCD/IT methods.

### 2.3. Data analysis

MS/MS spectra from both the *A. amphitrite* Sciex and Orbitrap files were assigned to the SNU_Aamp_1 proteome, GenBank GCA_009805615.1 [36] (electronic supplementary material, file S1). Spectra from the Orbitrap files were also assigned with both a reduced NRL transcriptomics database of only the identified adhesive proteins (Adhesive Database) [40] and a combination of the Adhesive Database and SNU_Aamp1 simultaneously (electronic supplementary material, files S3 and S4) in order to gain insight into sequences with low or no similarity between the databases. The *P. pollicipes* cement protein MS files (E4801_PPCIM1, E4802_PPCIM2 and E4803_PPCIM3) [33] were obtained from the Mendeley Data repository (http://dx.doi.org/10.17632/pgkf3mtb4m.1), and the spectra from these Orbitrap raw files were matched to the Ppol_2 proteome, RefSeq GCF_011947565.2 (electronic supplementary material, file S5).

MS/MS spectra of the *A. amphitrite* Sciex files were extracted to Mascot generic-formatted (MGF) peak lists by ProteoWizard (msconvert v. 3.0.19070) via automated scripting [41]. Mascot (v. 2.6.2, Matrix Science, Ltd, London, UK) and Scaffold (v. 4.8.9, Proteome Software, Portland, OR, USA) were used for peptide-spectrum matching (PSM) of the MGF files to the proteome database and an in-house list of 191 contaminant, mass standard, and reagent peptide sequences (e.g. trypsin, keratins, etc.) for a total of 296 sequences. Database search parameters included variable modifications previously described [35], less than three missed tryptic cleavage sites per peptide, and precursor and fragment ion tolerances set to +100 ppm and +0.6 Da, respectively.

MS/MS spectra of the Orbitrap raw files were assigned to the proteome databases using MaxQuant v. 1.6.10.43 (http://www.maxquant.org). Default settings were maintained with the following variations: variable modifications included oxidation (M), acetyl (Protein N-term) and the label-free quantification and match between runs features were enabled. For *P. pollicipes* samples, the additional modification of cysteine by MMTS (methylthiolation) was set.

The *A. amphitrite* Sciex wiff and MGF files are located on the ProteomeXchange Consortium via the PRIDE partner repository [42] with the dataset identifier PXD012730. The *A. amphitrite* Orbitrap raw files are available in the PRIDE partner repository with the dataset identifier PXD026105.

### 2.4. Sequence analysis

Sequence similarity between *A. amphitrite* and *P. pollicipes* proteins and all non-redundant proteins in the Protein Database Bank was assessed using NCBI BLASTp (www.ncbi.nlm.nih.gov/BLAST) [43] (electronic supplementary material, tables S1 and S2). Sequences were considered similar between the barnacle species at a threshold *E*-value > 0.0001. Conserved domains were identified using the NCBI Conserved Domain Database [44]. Protein amino acid composition was calculated using ExPASy ProtParam (http://web.expasy.org/protparam) [45]. *N*- and *O*-glycosylation sites were predicted using MusiteDeep [46].

Heatmaps and principal component analysis visualization were performed in R [47] using the function heatmap.2 with the packages gplots [48], factoextra [49] and ggplot2 [50].

## 3. Results

Here, data from previous publications that reported on the adhesive proteomes of *A. amphitrite* [35] and *P. pollicipes* [33] using transcriptomic-based information are reanalysed following the workflow outlined in electronic supplementary material, figure S1 using recently released genome assemblies for each species (*A. amphitrite*: SNU_Aamp_1, GenBank GCA_009805615.1 [36]; *P. pollicipes*: Ppol_2, RefSeq GCF_011947565.2).

**Figure 1.**
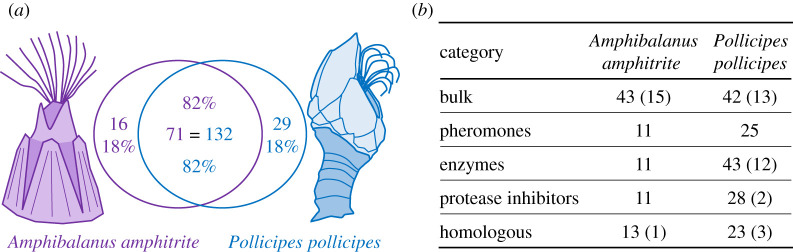
Comparison of the proteins identified via mass spectrometry in the adhesive of *A. amphitrite* and *P. pollicipes*. (*a*) Venn diagram indicating the number and percentage of the total identified proteins that do and do not share homology between the two species. Seventy-one *A. amphitrite* proteins align to 132 *P. pollicipes* proteins. (*b*) Number of proteins per protein category for each species, with the number of proteins with no homology to the other species in parenthesis. These broad categories are based on the annotation or conserved domains present in the protein sequences or homology to barnacle proteins identified in previous studies. The bulk proteins are either predicted to make up the bulk mass of the adhesive and may contribute to adhesion. Pheromones are either α-macroglobulins (settlement inducing or MULTIFUNCin pheromones) or have cupin domains (waterborne settlement pheromones). Enzymes cover a range of potential processes, including oxidation and protease (inhibition) activity. The homologous group includes the remaining proteins with homology to broader taxa. These numbers exclude highly likely intracellular proteins (35 *P. pollicipes*; three *A. amphitrite*), based on annotation and the lack of predicted signal peptides.

The adhesive proteomes identified through these methods are comprised of 87 *A. amphitrite* and 161 *P. pollicipes* proteins (electronic supplementary material, tables S3 and S4), excluding proteins involved in cellular processes (*A. amphitrite*: 3; *P. pollicipes*: 35). Cellular processes proteins likely entered the samples from cellular contamination and are not present in the native adhesive. The cellular processes proteins were identified by annotation as a protein with defined intracellular function and a lack of a signal peptide, unless the protein contained a signal peptide and a conserved motif that indicated its retention in the endoplasmic reticulum (i.e. HDEL). The 87 *A. amphitrite* adhesive proteins are mostly comprised of SNU_Aamp_1 sequences but also include some NRL transcriptomic-based sequences where warranted (seven sequences in the bulk protein category only). The homology of these proteomes was examined (figure 1*a*) to find proteins with a matching partner in the set of proteins identified in the adhesive proteome, rather than in all potential proteins encoded by the genomes. Seventy-one (82% of the total number identified) *A. amphitrite* and 132 (82%) *P. pollicipes* proteins have a match (*E*-value > 1 × 10^−4^) in the adhesive proteome of the opposite species. The identified proteins were divided into four broad categories: non-homologous bulk proteins (proteins unique to barnacles), pheromones, enzymes and remaining homologous proteins (proteins with homologous matches in non-Thoracican organisms) (figure 1*b* [parenthetical values indicate the number of unique proteins to the adhesive proteome of each species]).

### 3.1. Bulk proteins: overview

Here, the term bulk protein is used to describe any protein that is unique to thoracican barnacles. This category includes proteins such as CP19, CP43, CP52 and CP100 which have been proposed to either make up the structure or bulk of the adhesive or contribute directly to surface adhesion [8,14,23,51], although the distribution of some of these proteins beyond the surface interface indicates that they could have expanded physiological roles [52]. These proteins were named after their predicted molecular weight upon initial identification, followed by CP for cement protein [10,23,30], and this terminology has persisted as homologues in different species with different molecular weights identified. Here, Aa and Pp will be appended to the names of the proteins to identify the species. Transcriptomics of *A. amphitrite* [31] enabled proteomic analysis of the adhesive of *A. amphitrite* [14,21,35], resulting in an expansion in the number of potential bulk proteins. Bulk proteins were grouped based on sequence similarity into families with multiple members, where families were named after the molecular weight of one member. In this work, the bulk proteins include these families of previously described proteins and all other proteins in the adhesive proteome that do not have homology to non-Thoracica proteins in NCBI, following the approach taken by [33].

A similar number of bulk proteins were identified for *A. amphitrite* (43) and *P. pollicipes* (42) (figure 1*b*). Fifteen of the *A. amphitrite* and 13 of the *P. pollicipes* bulk proteins were unique to the adhesive proteome of each species. Two of the 15 unique *A. amphitrite* proteins and eight of the 13 unique *P. pollicipes* proteins had homologous matches in the entire genome of the other species, leaving 13 *A. amphitrite* and five *P. pollicipes* bulk proteins that are completely unique to each species.

### 3.2. Bulk proteins: families

Table 1 lists the accession numbers of the eight bulk protein families that have previously been described [14,35], along with sequence similarity metrics between the species. In some instances, BLAST alignment did not identify similar sequences in the *A. amphitrite* SNU_Aamp_1 proteome and the NRL transcriptomic-based proteome, or these matches were of low similarity. When this occurred, the original NRL transcriptomic-based sequences are included for analysis if these sequences were still identified when both the SNU_Aamp_1 and the Adhesive Database were used for spectral assignment. These instances are indicated when the accession is the protein name (example: AaCP43-1) rather than the SNU accession.

**Table 1.** Alignment of *A. amphitrite* SNU_Aamp1 cement protein families with the Ppol_2 proteome. Length: in amino acids; % ident: per cent identity; % cov: per cent coverage (Aamp sequences were used as the query during blast alignment); Amino acid %: percentage of amino acids in Ppol_2 protein sequences that had been identified as enriched in the homologous *A. amphitrite* proteins; accessions in bold contain a predicted signal peptide (SignalP > 0.5); accessions in italics indicate the sequence was not identified via mass spectrometry analysis of adhesive samples.

family	member	Aamp Accession	length	Ppol Accession	length	*E*-value	% ident	% cov
CP19	1	**KAF0308143.1**	203	**XP_037084082.1**	208	3 × 10^−9^	35%	83%
	like			XP_037073093.1	211	1 × 10^−6^	32%	57%
	2	KAF0313208.1	641	**XP_037079079.1**	134	9 × 10^−6^	42%	11%
	3	**KAF0308142.1**	456	**XP_037082465.1**	208	5 × 10^−8^	40%	36%
	4	KAF0308139.1	485	—	—	—	—	—
	5	**KAF0308140.1**	372	—	—	—	—	—
	6	KAF0308144.1	167	—	—	—	—	—
CP20	1	***KAF0308400.1***	129	—	—	—	—	—
	2	***KAF0288457.1***	118	—	—	—	—	—
CP34	1	**KAF0294702.1**	241	*XP_037087633.1*	3230	4 × 10^−21^	27%	89%
	3a	KAF0306042.1	193			2 × 10^−11^	27%	70%
	3b	KAF0295669.1	826			2 × 10^−13^	28%	37%
	3c	KAF0306041.1	749			4 × 10^−13^	28%	23%
	2	KAF0294701.1	196	—	—	—	—	—
	like	—	—	**XP_037084400.1**	454	—	—	—
CP43	like	***KAF0313428.1***^a^	500	**XP_037080313.1 XP_037080173.1**	449 449	3 × 10^−3^	30%	58%
	1	**AaCP43-1**	448			2 × 10^−19^	32%	86%
	2	KAF0306941.1	316			4 × 10^−26^	36%	90%
	3	KAF0295687.1	285	—	—	—	—	—
CP52	1a	**KAF0287842.1**	770	**XP_037074603.1**	356	2 × 10^−15^	39%	82%
	1b			**XP_037074712.1**	360	1 × 10^−14^	34%	30%
	1c	**KAF0311022.1**	388	**XP_037076617.1**	366	4 × 10^−89^	42%	96%
	1d			**XP_037076634.1**	320	2 × 10^−58^	41%	71%
	1e			**XP_037074597.1**	389	1 × 10^−21^	30%	80%
	2a	AaCP52-2	437	**XP_037084479.1**	225	3 × 10^−26^	44%	35%
	2b	KAF0312989.1	177	XP_037083850.1	257	5 × 10^−46^	63%	92%
	3a	AaCP52-3	536	**XP_037068781.1**	243	5 × 10^−19^	43%	70%
	3b	KAF0306294.1	127			6 × 10^−22^	43%	41%
CP57	1a	**KAF0312796.1**	541	—	—	—	—	—
	1b	**KAF0314286.1**	541	—	—	—	—	—
	3	KAF0293188.1^a^	247	—	—	—	—	—
	2	AaCP57-2	802	**XP_037075710.1**^a^	721	5 × 10^−108^	34%	89%
CP100	1	**KAF0310707.1**	1156	**XP_037084548.1**	1146	0	44%	96%
	2	**KAF0305748.1**	1000			0	38%	95%
CP105	1a	AaCP105-1	1558	XP_037082434.1	1202	1 × 10^−54^	25%	44%
	1b			XP_037088353.1	492	9 × 10^−39^	27%	31%
	1c			XP_037082049.1	425	5 × 10^−36^	29%	28%
	2a	AaCP105-2	1024	XP_037082494.1	1159	3 × 10^−162^	45%	65%
	2b	KAF0305740.1	178	XP_037083173.1	314	4 × 10^−25^	52%	48%
	3a	AaCP105-3	545	**XP_037082052.1**	495	9 × 10^−30^	28%	48%
	3b	**KAF0305741.1**	407			2 × 10^−12^	24%	44%

^a^Duplicated genes or entries exist in either SNU_Aamp1 or Ppol2; shading indicates members of the same family (based on sequence similarity >1 × 10^−4^), with darker or alternate colours delineating distinct families.

After identifying the most similar matches in the SNU_Aamp_1 proteome to the NRL transcriptomic-based sequences, a self-BLAST was performed to verify that family members had sequence similarity as before. In most cases, sequence similarity was maintained in families, but three families (AaCP43, AaCP52 and AaCP57) had at least one member that no longer fit. These instances are denoted by greyed shading in table 1.

Five of the *P. pollicipes* cement protein families show fewer family members than analogous families in *A. amphitrite*. For example, the *A. amphitrite* CP19 family contains six proteins, while the *P. pollicipes* CP19 family contains four proteins. The sequence similarity between the CP19 proteins is low (*E*-value > 1 × 10^−5^), and the six AaCP19 proteins were each identified as individual proteins, but PpCP19-1, -2 and -3 clustered together as one entry because the sequences and associated MS data were indistinguishable (electronic supplementary material, table S4). All other cement proteins for both species (besides AaCP34-3a, b and c) were identified as individual proteins and not clusters. CP20 homologues were not identified in *P. pollicipes*, even when using AaCP20 sequences from NCBI (AFX74689.1, AFX74690.1 and AFX74691.1). All of the AaCP34 proteins, which are 193–826 amino acids in length, had sequence similarity to a single large *P. pollicipes* protein (XP_037087633.1, 3230 amino acids) encoded by the Ppol_2 genome; however, this protein was not identified via MS. A different protein (XP_037084400.1), which had sequence similarity to XP_037087633.1 but not to the AaCP34 proteins, was identified in the *P. pollicipes* adhesive. The AaCP57 proteins are split into two families, one with proteins that matched to AaCP57-1 and -3, and the original AaCP57-2 sequence as its own family. No matching sequence for AaCP57-2 was identified in SNU_Aamp_1, but this sequence has high homology to the *P. pollicipes* protein XP_037075710.1 (*E*-value = 5 × 10^−108^). Finally, the AaCP100 family of two proteins is collapsed into one *P. pollicipes* protein with high sequence similarity between the two species.

The CP52 protein family is the only family to show a larger number of proteins in *P. pollicipes* than in *A. amphitrite*, and further, the structure of the AaCP52 family is significantly altered after inclusion of the SNU sequences. Four SNU AaCP52 proteins were identified, but the alignment between the NRL transcriptomics sequences for AaCP52-2 and -3 was low, so these sequences were also included. The homology between all AaCP52 sequences is too low to support containing these proteins in one family; as such, CP52 is split into three families (AaCP52-1, -2 and -3). The AaCP52-1 family has only two proteins, while the PpCP52-1 family contains five. The other two CP52 families have similar numbers of proteins (1–2).

The AaCP43 family also underwent extensive protein sequence rearrangement in comparison with its previous descriptions [14,35] after considering the SNU protein sequences. AaCP43-1 is a protein that was first identified using HFIP (hexafluoroisopropanol) as a solvent and its sequence was verified via RT-PCR sequencing (mRNA sequence KY285984.1) [14]. When searching for the matching sequence to AaCP43-1 in SNU_Aamp_1, two entries with only 50% sequence similarities were identified (KAF0313428.1 and KAF0306452.1), neither of which was identified via MS. The AaCP43-1 mRNA coding sequence was used to search the *A. amphitrite* genome (SNU_AA5) for the presence of a similar genomic region (electronic supplementary material, figure S2). A region on Contig787 with 100% coverage and 95% sequence similarity was identified (electronic supplementary material, figure S2*a*). This region has no predicted coding sequence in the NCBI assembly (electronic supplementary material, figure S2*b*), but the six-frame translation reveals two coding sequences with an approximately 100 nucleotide gap in between (electronic supplementary material, figure S2*c*). The protein translations of these two truncated sequences were aligned with AaCP43-1 (electronic supplementary material, figure S3). The first sequence aligns along the N-terminal of AaCP43-1 with low similarity (*E*-value = 0.002, 43% identity, 13% coverage), and the second sequence aligns after a gap of approximately 40 amino acids to the C-terminus with high similarity (*E*-value = 2 × 10^−153^, 87% identity, 67% coverage), including a region of approximately 250 amino acids with almost 100% consensus. Sequence 2 and AaCP43-1 were both identified via MS; therefore, NRL transcriptomics sequence of AaCP43-1 was included for further analysis. Other NRL transcriptomics sequences for AaCP43 family members align either to different regions of the same protein or to no SNU proteins (electronic supplementary material, table S1). Additionally, AaCP43-3 (KAF0295687.1) shows low sequence similarity to the other AaCP43 family members and should be considered an unrelated protein. Two nearly identical PpCP43 proteins align to the AaCP43 proteins, but both proteins were identified individually, and not clustered, in the MS results.

The other bulk proteins identified (11 *A. amphitrite*, 19 *P. pollicipes*) do not align with any of the previously published cement protein families. A group of four proteins (*A. amphitrite*: KAF0303467.1 and KAF0314143.1; *P. pollicipes*: XP_037072930.1 and XP_037072849.1) exhibit sequence similarity to each other and could be classified as a novel family. Of these other bulk proteins, four *A. amphitrite* and seven *P. pollicipes* proteins exhibit some sequence similarity to a bulk protein in the adhesive proteome of the other species, while five *A. amphitrite* and 11 *P. pollicipes* proteins do not.

### 3.3. Bulk proteins: amino acid composition

**Figure 2.**
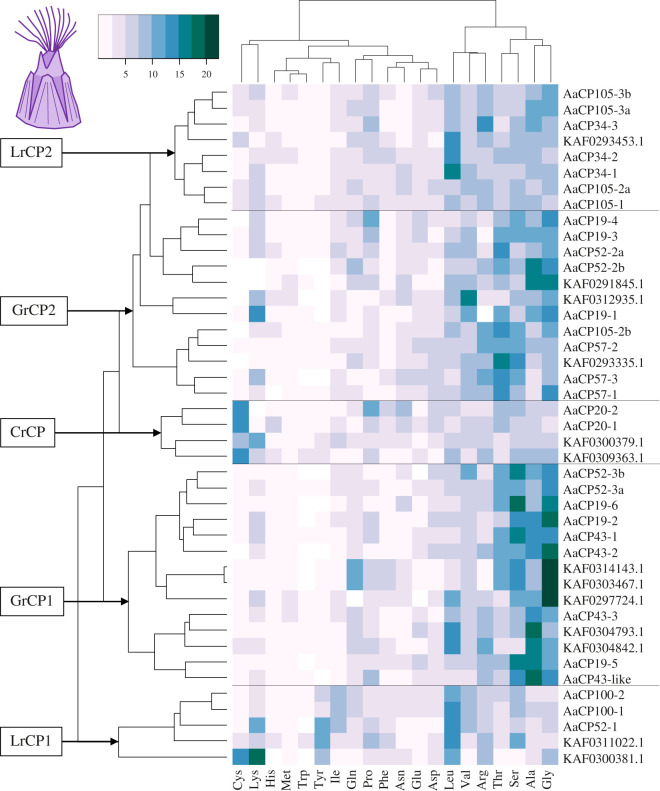
Heatmap of *A. amphitrite* bulk cement protein amino acid (AA) composition. All bulk cement proteins are included in this analysis. Cells are coloured by amino acid percentage. Clustering is based on row means and shows distinct grouping of proteins based on amino acid enrichment. Five distinct groups of cement proteins are revealed by clustering: two leucine-rich groups (LrCP1 and LrCP2), two glycine-rich groups (GrCP1 and GrCP2) and one cysteine-rich group (CrCP). CP, cement protein.

**Figure 3.**
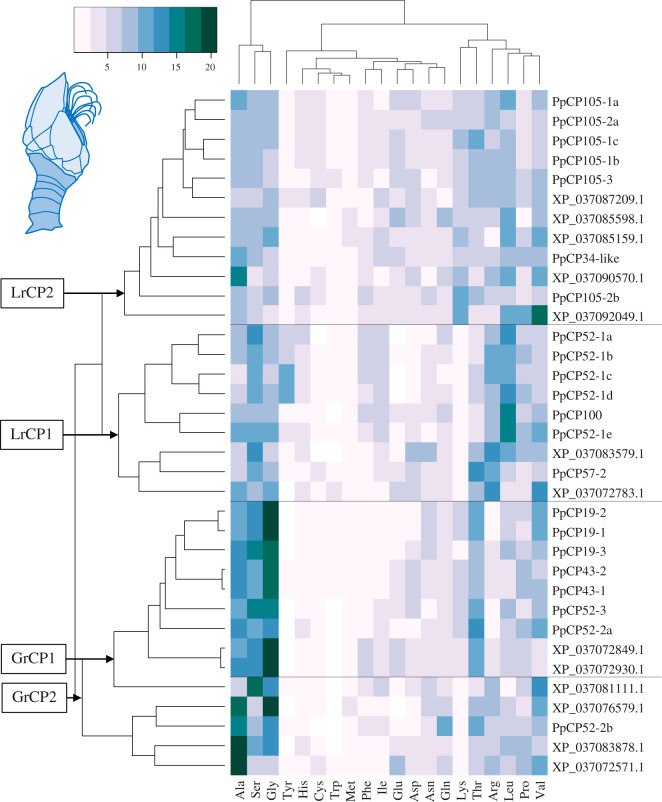
Heatmap of *P. pollicipes* bulk cement protein amino acid (AA) composition. Only glycine and leucine-enriched bulk cement proteins are included in this analysis. Clustering is based on row means and shows distinct grouping of proteins based on amino acid enrichment. The two leucine-rich groups (LrCP1 and LrCP2) glycine-rich proteins (GrCP1 and GrCP2) groups seen in *A. amphitrite* are apparent in this *P. pollicipes* analysis.

Heatmaps were created to analyse how all bulk proteins (the cement proteins listed in table 1 and the other identified non-homologous proteins) cluster based on total amino acid composition (*A. amphitrite*: figure 2; *P. pollicipes*: figure 3), as barnacle cement proteins have also been classified by amino acid biases [18,33,35]. The proteins in the heatmaps form clusters of proteins with enrichment for leucine (Leucine-rich Cement Protein: LrCP), glycine, alanine, serine and threonine (Glycine-rich Cement Protein: GrCP), and an additional cluster for *A. amphitrite* bulk proteins with enriched cysteine content (Cysteine-rich Cement Protein: CrCP).

The column dendrogram for *A. amphitrite* shows three groupings of amino acids that drive protein clustering: cysteine and lysine; leucine, valine and arginine; and glycine, alanine, serine and threonine. The *A. amphitrite* bulk proteins separate into five major clusters, LrCP1/2, GrCP1/2 and CrCP (figure 2). AaLrCP and AaGrCP both separate into two distinct clusters. AaLrCP1 proteins exhibit further enrichment with tyrosine and isoleucine, while AaLrCP2 proteins have elevated arginine, alanine and glycine. Both AaGrCP1 and -2 proteins generally have high levels of glycine, alanine, serine and threonine, or some combination thereof, but the profiles of these two clusters do not appear to differ greatly beyond AaGrCP1 members having higher levels of overall enrichment.

The *P. pollicipes* bulk proteins separate into two major (PpLrCP and PpGrCP) and three minor clusters (electronic supplementary material, figure S4). The eight proteins forming the minor clusters (enriched for: proline; threonine and valine; glycine and arginine) were removed from further analysis for simplicity and the heatmap was remade (figure 3). No cysteine-enriched cluster is observed in *P. pollicipes*. PpLrCP and PpGrCP groups show some enrichment for alanine, serine and glycine, while this enrichment is only seen in the AaGrCP groups. PpLrCP and PpGrCP can be further divided into two separate clusters. The PpLrCP1 proteins mostly display enrichment for leucine and arginine, with some apparent enrichment for alanine, serine and glycine as well. The PpLrCP2 proteins show little enrichment for any amino acid overall as a group. The PpGrCP1 proteins show an abundance of alanine, serine and glycine and some enrichment of threonine. The PpGrCP2 proteins mostly show enrichment for alanine and glycine, with little to no enrichment for either serine or threonine. The column dendrogram provides support for alanine, serine and glycine driving clustering of *P. pollicipes* bulk proteins.

**Figure 4.**
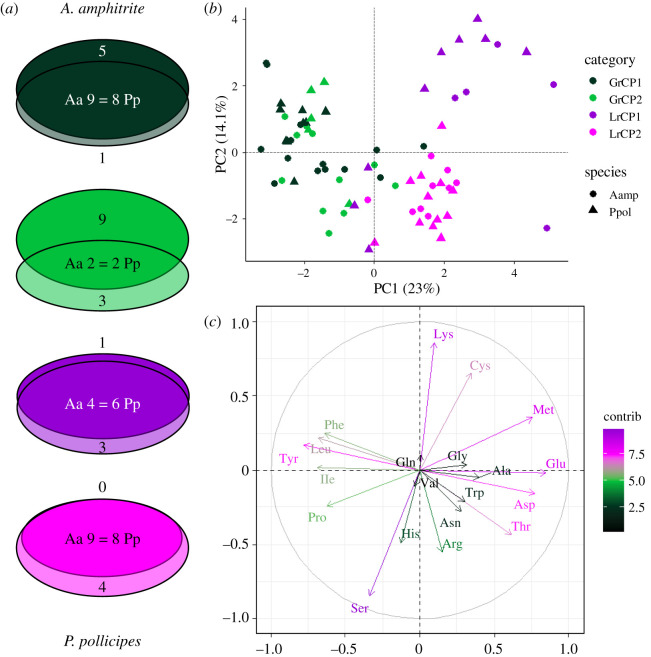
Comparison of *A. amphitrite* and *P. pollicipes* bulk glycine (GrCP) and leucine (LrCP)-rich cement protein. (*a*) Venn diagrams showing overlap between the *A. amphitrite* and *P. pollicipes* GrCP and LrCP groups. The overlapping proteins do not contain the same number of proteins, as a single protein from one species can align significantly to multiple proteins of the other species; therefore, these dissimilar values are indicated by *A. amphitrite* (Aa) proteins = *P. pollicipes* (Pp) proteins. Colour key is that same as in *b*. (*b*) Principal component analysis (PCA) of individual proteins from both *A. amphitrite* (Aamp, circles) and *P. pollicipes* (Ppol, triangles) with GrCP and LrCP categories indicated by colour. (*c*) PCA variable plot of the amino acids driving protein separation seen in (*b*). Amino acid vectors are coloured by contribution score.

The overlap between similar *A. amphitrite* and *P. pollicipes* proteins in the GrCP and LrCP groups is visualized as Venn diagrams (figure 4*a*). The GrCP1 proteins overlap extensively (although five *A. amphitrite* proteins do not have a matching *P. pollicipes* partner), while the GrCP2 proteins show little overlap. AaLrCP1 and -2 proteins nearly align with *P. pollicipes* proteins in the same category (only one AaLrCP1 protein does not), yet *P. pollicipes* has several unique proteins in each group. Multiple instances exist where proteins with sequence similarity between the species appear in different amino acid groups. The analysis of proteomes permits us to broaden protein sets when comparing homology between gooseneck and acorn barnacle adhesive proteins beyond previous work [13]. Generally, *P. pollicipes* was observed to have low protein sequence alignment with other acorn barnacles ranging from 26 to 36% looking across just two proteins, cp19 k and cp100 k. Homology was higher among acorn barnacle species in the case of cp19 k, cp20 k and cp100 k (ranging mostly between 18 and 45%, with cp100 k occupying 42–45% and outliers reaching 60 and 64%). In our analysis, protein identity ranges between 24 and 63% with CP100 sequences ranging from 38 to 44% (table 1) identity. Based on per cent identity, higher overall homology was observed between *P. pollicipes* and *A. amphitrite* proteins sequences derived from genomic sequences than previous analysis, largely falling between 30 and 45% with outliers at 52 and 63%. These values relate *P. pollicipes* with *A. amphitrite* at homology values similar to previous comparisons among acorn species.

The amino acid composition of *A. amphitrite* and *P. pollicipes* LrCP and GrCP groups was further analysed with principal component analysis (PCA). PC1 explains most of the separation between the LrCP and GrCP groups (figure 4*b*; the same plot but with individual protein names is provided as electronic supplementary material, figure S5). The GrCP1 and -2 groups overlap while the LrCP1 and -2 groups are mostly separated, although one *A. amphitrite* (KAF0300381.1) and three *P. pollicipes* (XP_037083579.1, PpCP57-2 and XP_037072783.1) proteins do not cluster with the other LrCP1 proteins. These outlier proteins also form distinct nodes in the amino acid heatmaps (figures 2 and 3). No separation by species is clearly observed. A variable plot of the PCA data (figure 4*c*) shows that glycine, serine and alanine contribute the greatest amount to the location of the GrCPs, while leucine and tyrosine contribute to the location of the LrCPs. Multiple amino acid appear to contribute to the separation of the LrCP1 and -2 groups.

### 3.4. Bulk proteins: feature characterization

The barnacle bulk proteins show no sequence similarity to other proteins in NCBI, and few conserved domains are observed. KAF0304842.1 (AaGrCP1) and XP_037090570.1 (PpLrCP2) align with higher similarity than most of the bulk proteins (*E*-value = 2 × 10^−46^, 59% identity), and both contain a predicted C-type lectin domain (cd00037). KAF0312935.1 (AaGrCP2) and XP_037085159.1 (PpLrCP2) align with lower similarity (*E*-value = 3 × 10^−15^, 35% similarity) and both contain a predicted juvenile hormone-binding protein domain (cl12117). Eight *P. pollicipes* proteins have predicted annotations (XP_037068781.1, XP_037081766.1, XP_037073093.1, XP_037093877.1, XP_037081111.1, XP_037078034.1, XP_037079857.1, XP_037070787.1), but little support for these were observed after blast analysis.

Cement proteins are hypothesized to reach the surface interface either via transport through a series of ducts after being produced in cement glands [53–56] and/or secretion from a layer of epithelial cells at the leading edge of the barnacle base that also likely contribute to cuticle formation in acorn barnacles, as the interface building process is complex and intimately related to moulting [16,17]. Either way, the proteins would be secreted from their origin cells and contain a signal peptide sequence. Approximately 65% of the *A. amphitrite* and 75% of the *P. pollicipes* bulk proteins contain predicted signal peptides (table 1 and electronic supplementary material, tables S1 and S4). The majority of the *P. pollicipes* bulk proteins with no predicted signal peptide are PpCP105 family members. No clear patterns for *A. amphitrite* bulk protein signal peptides were observed as a function of family or amino acid category.

**Figure 5.**
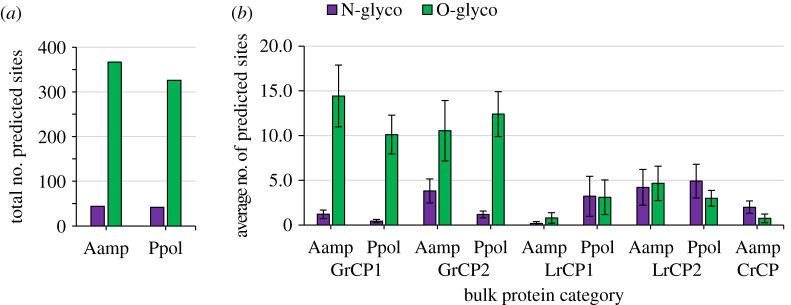
Glycosylation site prediction of bulk cement proteins. (*a*) Total number of predicted *N*- and *O*-linked glycosylation sites for *A. amphitrite* (Aamp) and *P. pollicipes* (Ppol) bulk adhesive proteins. (*b*) Average number of *N*- and *O*-linked glycosylation sites for each amino acid group (glycine rich: GrCP1 and GrCP2; leucine rich: LrCP1 and LrCP2; cysteine rich: CrCP).

Finally, the potential for bulk protein glycosylation was examined (figure 5). The number of predicted glycosylation sites for overall bulk proteins (figure 5*a*) and categories (figure 5*b*) is similar between species. Overall, approximately 40 *N*-linked and >300 *O*-linked glycosylation sites are predicted for all bulk proteins of each species. Both *A. amphitrite* and *P. pollicipes* GrCP groups are predicted to have on average 10–15 *O*-linked sites compared with fewer than four *N*-linked sites per protein. LrCP groups are predicted to have an even lower number of *N*- and *O*-linked sites per protein (less than five). The AaCrCP group has a low number of predicted sites for either type of glycosylation. The minor clusters of *P. pollicipes* bulk proteins that show enrichment for either proline or threonine and valine also have a high number of predicted *O*-linked (12–27) and a low number of predicted *N*-linked (less than four) glycosylation sites (data not shown), similar to the GrCP groups.

### 3.5. Pheromones

**Figure 6.**
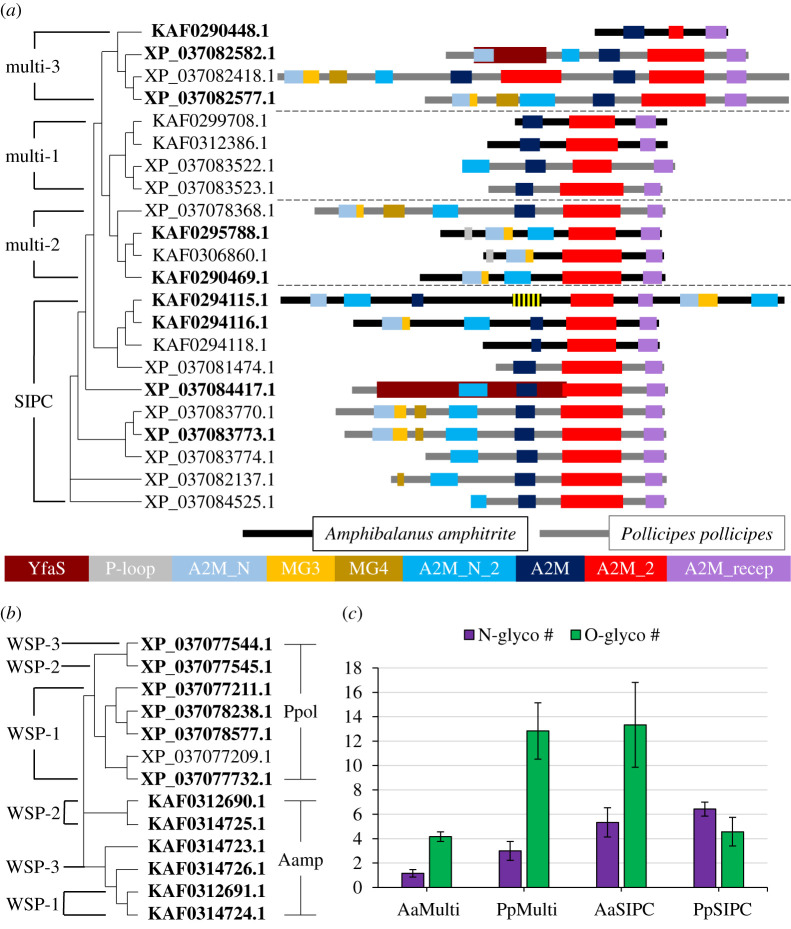
Multiple sequence alignment of the α-macroglobulin and waterborne settlement pheromones identified in the adhesive. (*a*) A tree constructed from multiple sequence alignment data of the α-macroglobulin pheromones that contain an Isopren_C2_like domain shows clustering by family (Multi: MULTIFUNCin; SIPC: settlement inducing complex). Bolded accession numbers indicate sequences with predicted signal peptides. The conserved domains identified for each protein are shown to the right. *A. amphitrite* proteins are distinguished by a black backbone (KAF accessions) and *P. pollicipes* are distinguished by a grey backbone (XP accessions). One protein (KAF0294115.1) contains an Rnase_HI_RT_non_LTR (cd09276; indicated by a yellow and black striped box) that was likely inserted by a transposon. Dashed lines between families were added to improve visualization. (*b*) Multiple sequence alignment of all waterborne settlement pheromones (WSPs). The WSPs cluster by species rather than by family, as seen in the α-macroglobulin pheromones. Each of these proteins contains the domain cupin_RmlC-like super family (cl40423). Bolded accession numbers indicate sequences with predicted signal peptides. (*c*) The average number of predicted *N*- and *O*-linked glycosylation sites for the α-macroglobulin pheromones. The MULTIFUNCin familes (Multi-1 through 3) have been combined for analysis. AaMulti: *A. amphitrite* MULTIFUNCin; PpMulti: *P. pollicipes* MULTIFUNCin; AaSIPC: *A. amphitrite* settlement inducing complex; PpSIPC: *P. pollicipes* settlement inducing complex.

Two major types of pheromones have been characterized in barnacles: the α-macroglobulin pheromones (settlement inducing protein complex: SIPC; MULTIFUNCin: Multi) [57–59] and the waterborne settlement pheromones (WSP) [60,61]. Both types have been identified in the adhesive of *A. amphitrite* and *P. pollicipes* previously [33,35] and in this study (figure 6).

In addition to serving as cues for juvenile settlement and appearing in the adhesive of diverse marine invertebrates [7], α_2_-macroglobulins are conserved actors of the innate immune system that function to clear circulating proteases [62]. This molecular function relies on a set of specific conserved domains (A2M_N, MG3, MG4, A2M_N_2, A2M, A2M_2/Isopren_C2 and A2M_recep), where the A2M_N_2 domain (pfam07703) serves as a bait region for cleavage by proteases. Upon cleavage, α_2_-macroglobulins undergo a conformation change where a thiol ester bond is formed between the A2M_2 domain (cl08267) and the protease, resulting in a cage-like entrapment and inactivation of multiple classes of proteinases. The conformational change after cleavage also exposes a conserved COOH-terminal receptor-binding domain in the A2M_recep domain (pfam07677) which targets the protein for degradation [63,64].

A distance tree of the α-macroglobulin pheromones containing a specific conserved domain (A2M_2/Isopren_C2_like, cl08267) shows that the proteins separate into four distinct families (SIPC and Multi 1–3), with members from each species clustering together (figure 6*a*). In the MS results, eight separate *A. amphitrite* α-macroglobulin pheromones were identified, although one SIPC entry contains a cluster of six separate proteins. The major protein making up this cluster (KAF0294116.1, 1450 amino acids) is similar to other reported sequences of SIPC (AAR33079.1, 1547 amino acids, 90.74% identity; AMR58954.1, 1533 amino acids, 90.32% identity). Of the 14 total *A. amphitrite* α-macroglobulin protein sequences identified, nine contained the A2M_2 domain. Twenty-two individual *P. pollicipes* α-macroglobulin proteins were identified, but only 13 contained the A2M_2 domain and were included in this analysis. Of the 22 A2M_2 domain containing proteins from both species, nine have a predicted signal peptide. The *A. amphitrite* and *P. pollicipes* A2M_2 domain α-macroglobulins exhibit high sequence similarity to each other (electronic supplementary material, table S4: *E*-value > 1 × 10^−175^). The α-macroglobulin pheromones contain different combinations of approximately 10 predicted conserved domains. Most of the A2M_2 domain containing proteins also have A2M (pfam00207) and A2M_recep (pfam07677) domains. The Multi-1 family—comprised of proteins from both species—are all similar as they contain these three core domains (along with one A2M_N_2 domain, pfam07703 in XP_037083522.1), are approximately 700–1000 amino acids long, and do not have predicted signal peptides. The Multi-2 and -3 proteins are more diverse and show little similarity between the species beyond the three core domains. The AaMulti-2 proteins do not contain the A2M domain (the single PpMulti-2 protein does), but two contain a P-loop domain (cl38936), not observed elsewhere among the α-macroglobulin proteins. The Multi-2 proteins most consistently contain A2M_N (pfam01835), MG3 (pfam17791) and A2M_N_2 domains compared with all other α-macroglobulin pheromones. AaMulti-3 contains only the three core domains, with a reduced Isopren_C2_like domain, while the PpMulti-3 proteins are at least double the length and contain a combination of other domains. The AaSIPC KAF0294116.1 structure is similar to six of the PpSIPC proteins. Some *P. pollicipes* α-macroglobulin proteins contain YfaS (COG2373) and MG4 domains (pfam17789), which are not observed in the *A. amphitrite* α-macroglobulin proteins.

The relationship between the *A. amphitrite* and *P. pollicipes* WSP proteins is dissimilar to the relationship of the α-macroglobulin proteins (figure 6*c*). Here, the distance tree indicates that the proteins separate by species rather than WSP family, which could be indicative of the protein's species-specific recruitment activity. All WSP proteins are 178–265 amino acids long, contain only cupin_RmlC-like domain (cl40423), and all but one have predicted signal peptides. The WSP proteins also have lower sequence similarity between the species than the α-macroglobulin proteins (electronic supplementary material, table S4: *E*-value = 1 × 10^−48^ – 1 × 10^−75^), but this similarity is higher than that exhibited between many of the bulk proteins.

As glycosylation of SIPC has been documented [65], the predicted glycosylation patterns for all of the pheromones were examined (figure 6*d*). The WSP proteins have negligible potential for glycosylation (less than three sites per protein), while the α-macroglobulin proteins have higher levels of potential glycosylation (eight *N*-linked and 19 *O*-linked predicted sites per protein maximum). The locations of predicted *N*-glycosites for AaSIPC (KAF0294116.1) and PpSIPC (XP_037083773.1) were compared to seven previously predicted *N*-glycosites of *A. amphitrite* SIPC (AAR33079.1, data not shown) [59]. The six AaSIPC *N*-glycosites aligned exactly with those of AAR33079.1, while the region that contains the seventh AAR33079.1 *N*-glycosite is missing in AaSIPC. For PpSIPC, four of its eight total predicted *N*-glycosites aligned with those of AAR33079.1. Generally, for all of the α-macroglobulin proteins, the SIPC family for both species have more potential *N*-linked sites than the MULTI proteins families. Finally, PpMulti and AaSIPC contain more *O*-linked sites than the PpSIPC and AaMulti proteins.

Family and species differences exist in the location of the predicted glycosylation sites in relation to the conserved domains (electronic supplementary material, table S5). The Isopren_C2_like domain contains 1–3 predicted *N*-linked sites in the SIPC and PpMulti-1 family members, but either no or one *O*-linked site in the AaMulti-1 and Multi-2 and -3 families. The PpSIPC A2M domain contains 1–2 predicted *N*-linked sites, while the AaSIPC family has no potential glycosylation in this domain. The reverse pattern is seen for the A2M_recep domain, where the AaSIPC proteins contain two *O*-linked sites and the PpSIPC proteins contain almost none (one *N*-linked site for one protein). Predicted glycosylation sites exist for other domains, but the pattern is less clear.

### 3.6. Enzymes and protease inhibitors

Many of the identified adhesive proteins likely function as enzymes or protease inhibitors. All of the 11 enzymes and 11 protease inhibitors in the *A. amphitrite* adhesive have a matching partner in the *P. pollicipes* adhesive, but the converse is not true: 12 of the 43 *P. pollicipes* enzymes and two of the 28 *P. pollicipes* protease inhibitors are unique. The enzymes identified in the adhesive fall into 10 families with an array of potential functions (table 2). Enzymes with potential oxidase (peroxidases and lysyl oxidases) and serine protease functions are found in both species, but more members of the peroxidases (10 versus four) and serine proteases (22 versus five) are identified in *P. pollicipes*. In addition, several enzymes covering a range of processes were identified in *P. pollicipes* and not in the adhesive of *A. amphitrite*, including a cyclophilin and enzymes involved in cellulose and chitin degradation (lytic polysaccharide mono-oxygenase (LPMO), chitin deacetylase and chitinase). Five classes of potential protease inhibitors were identified: serine protease inhibitors (serpins, Kunitz and pacifastin), peptidase inhibitors and cysteine protease inhibitors. All types except for the pacifastin serine protease inhibitors (unique to *P. pollicipes*) were identified in both species. More serpin and peptidase inhibitors were identified in *P. pollicipes* (15 versus four and nine versus four, respectively). The majority of members of protease inhibitor families have predicted signal peptides for both species, although more of the *P. pollicipes* than the *A. amphitrite* enzyme members do.

**Table 2.** Enzyme and protease inhibitor families identified in the adhesive proteomes of *A. amphitrite* and *P. pollicipes*.

family	# Aamp	# Ppol	conserved domains	accession
peroxidase	4 (3)	10 (6)	An_peroxidase	pfam03098
lysyl Oxidase	2 (1)	2 (2)	Lysyl_oxidase	cl03127
			SR	smart00202
serine protease	5 (1)	22 (18)	Tryp_SPc	cd00190
			CLIP_1	pfam18322
			Tryp_SPc super family	cl21584
cyclophilin	0	1 (1)	cyclophilin super family	cl00197
cellulase	0	1 (0)	LPMO_10	pfam03067
chitin deacetylase	0	3 (3)	CE4_CDA_like_1	cd10974
			LDLa	cd00112
			CBM_14	pfam01607
chitinase	0	4 (3)	CBM_14	pfam01607
			GH18_chitinase-like super family	cl10447
serine protease inhibitor (serpin)	4 (3)	15 (12)	serpin	cd00172
			serpin super family	cl38926
			Asp super family	Cl37951
			serpin_crustaceans…	cd19594
pacifastin serpin	0	1 (1)	Pacifastin_I	pfam05375
Kunitz serine protease inhibitor	2 (2)	1 (1)	KU super family	cl00101
peptidase inhibitor	4 (2)	9 (6)	WAP	PF00095
cysteine protease inhibitor	1 (1)	2 (1)	Thyroglobulin_1	pfam00086
			TY	cd00191
			TY super family	cl00150

# *Aamp* & # *Ppol*: total number of proteins identified in each family for *A. amphitrite* and *P. pollicipes* and (the number of proteins with predicted signal peptides); conserved domains and their accessions as identified via the NCBI Conserved Domains Database.

### 3.7. Homologous proteins

The remaining proteins that could not be classified as bulk proteins, pheromones or enzymes are classified here as homologous proteins. These proteins have homologues seen in a wider range of taxa than the bulk proteins, and many contain predicted conserved domains that imply a biological function. *A. amphitrite* has 13 and *P. pollicipes* has 23 homologous proteins. Only one *A. amphitrite* (a mucin type protein) and three *P. pollicipes* homologous proteins (putative defense, fasciclin and c-type lectin proteins) are unique to their species.

The families and potential functions of the homologous proteins identified in the adhesive are listed in table 3. The homologous proteins form smaller families than the enzymes and protease inhibitors, containing no more than six members. The majority of the homologous proteins also contain predicted signal peptides. The protein families can be further grouped by potential functions. The hemocytin/mucin [66], apolipophorin [67], vitellogenin [68], SVWC (single domain von Willebrand factor type C) [69], putative defense protein [70], C-type lectin [71] and CAP (cysteine-rich secretory proteins, antigen 5 and pathogenesis-related 1 proteins) families are all implicated in arthropod innate immunity by the promotion of pathogen agglutination, although each protein could also perform other biological functions, including lipid transport involved with reproduction. The fasciclin [72] family has a potential broad adhesion function, while the remaining families likely function with a more specialized binding capacity (proteoglycans: glycoprotein with carbohydrate and calcium binding; annexin: phospholipid binding [73]; acetylcholine receptor: acetylcholine binding; cuticle proteins: chitin binding).

**Table 3.** Homologous protein families identified in the adhesive proteomes of *A. amphitrite* and *P. pollicipes*.

family	# Aamp	# Ppol	putative function	# N-glyco	# O-glyco
hemocytin/mucin	4 (1)	1 (1)	immunity	7; 11	68; 65
apolipophorin	1 (1)	3 (1)	immunity	4; 13	7; 9
vitellogenin	2 (1)	1 (1)	immunity	2; 9	4; 3
SVWC	1 (1)	1 (1)	immunity	0; 1	0; 2
putative defense protein	0	1 (1)	immunity	NA; 0	NA; 4
C-type lectin	0	1 (1)	immunity	NA; 2	NA; 6
CAP	2	2 (2)	immunity	1; 0	11; 7
fasciclin	1	2 (1)	adhesion	3; 9	2; 4
proteoglycan	1 (1)	3 (2)	glycoprotein	23; 3	40; 45
annexin	1 (1)	2 (2)	phospholipid binding	3; 2	25; 19
acetylcholine receptor	1 (1)	1 (1)	ligand binding	2; 1	0; 0
cuticle protein	1 (1)	6 (6)	chitin binding	0; 1	0; 9

Family acronyms: SVWC, single-domain von Willebrand factor type C; CAP, cysteine-rich secretory proteins, antigen 5 and pathogenesis-related 1 proteins.

# Aamp & # Ppol: total number of proteins identified in each family for *A. amphitrite* and *P. pollicipes* and (the number of proteins with predicted signal peptides).

# N-glyco & # O-glyco: the maximum number of predicted N- and O-linked glycosylation sites for each family; *A. amphitrite* #; *P. pollicipes* #.

While many of the same types of homologous proteins are observed in the adhesive proteome of both *A. amphitrite* and *P. pollicipes*, the conserved domains and signal peptide presence in some families differ between the species. The hemocytin proteins of each species are predicted to have a variety of conserved domains with most present in both species; the AaHemocytins do not have a predicted signal peptide, while the PpHemocytin protein does. The *A. amphitrite* proteins identified with a CAP domain (cd05380; found in a wide array of proteins) also lack a predicted signal peptide, while the *P. pollicipes* proteins have one. In addition, the PpCAP proteins have an MAM super family (meprin, A5 protein, and protein tyrosine phosphatase Mu, cl27660) domain, which is an adhesive extracellular domain found in transmembrane proteins [74]. Each species has a protein with the predicted fasciclin domain (pfam02469; involved in adhesion), though the AaFasciclin protein only contains one, while the PpFasciclin contains four. The *P. pollicipes* proteoglycan proteins contain a number of immunoglobulin-like and lipoprotein domains that are not present in the *A. amphitrite* homologues. The annexin proteins in both species contain four annexin domains (pfam00191; involved in adhesion) with the PpAnnexin proteins containing an additional CLIP_1 domain (pfam18322; interacts with serine protease-like domains). Overall, the *P. pollicipes* homologous proteins tend to have higher rates of predicted signal peptides and more diverse conserved domains.

Analysis of the predicted glycosylation sites for the homologous protein families indicates that while many show low levels of potential glycosylation, several have the potential for high levels, including members of the hemocytin (*N*: 11; *O*: 68), apolipophorin (*N*: 13; *O*: 9), proteoglycan (*N*: 23; *O*: 45) and annexin (*N*: 3; *O*: 25) families. The number of predicted glycosylation sites does not vary greatly between species.

## 4. Discussion

Barnacles have evolved a sessile existence dependent on their ability to adhere to surfaces using a proteinaceous adhesive. This adhesive does not rely on the same adhesion mechanisms described in other marine invertebrates [22,23]. Barnacle adhesive is composed of both novel proteins without homology to non-Thoracican organisms and a number of proteins with a predicted biological function that are highly conserved. How these proteins contribute to adhesion remains poorly understood. A more thorough characterization of the proteins present in the adhesive across the barnacle tree of life can provide valuable insight into how the components of this robust adhesive have been both preserved and diversified during evolution. The development of improved sample processing techniques for proteomics analysis [33–35] and genomic assemblies for acorn [36] and stalked (Ppol_2, RefSeq GCF_011947565.2) barnacles now allow for more in-depth analyses that go beyond general observations, enabling the comparison of proteins present at the adhesive interface across species in unprecedented detail.

Early studies of barnacle adhesive relied on gel-based analysis and led to the discovery of a handful of non-homologous proteins [10,23,30]. More recently, better breakdown of the adhesive combined with MS analysis has revealed that the adhesive is composed of potentially hundreds of proteins [14,33–35], some of which are unique to barnacles while others are not and likely participate in a number of biological activities at the substrate interface, such as moulting, immunity, conspecific communication and, broadly, adhesion. Employment of various imaging and spectroscopic techniques at the acorn barnacle substrate interface has revealed a complex and dynamic area capable of clearing microbial biofilms, preparing the surface for adhesion, and secretion and delivery of the adhesive chemistries, all progressing through a cyclic process of growth and expansion interrelated with moulting [16,17,75,76].

One major advantage of having genomic rather than transcriptomic-derived protein sequences is that complete coding sequences allow for signal peptide analysis of the identified proteins. This analysis provides more definitive characterization of proteins involved in intracellular processes that are most likely not present in the native adhesive and perhaps introduced during sample collection. Our analysis reveals many of the identified proteins that lack obvious intracellular roles still do not contain signal peptides, raising the question of whether proteins lacking a signal peptide that would facilitate their transport to the substrate interface are the result of contamination or presented through a different means during barnacle growth. We note that many cellular structural components, including actin, tubulin, myosin, etc., were identified in the *P. pollicipes* adhesive analysis but not in *A. amphitrite*, which may indicate that the identified proteins without signal peptides that do not have an attributed intracellular role may be present in the native barnacle adhesive. These proteins could be released from epithelial cells known to be present along the acorn barnacle basal leading edge [17]. While the direct observation of such a cell layer has not been noted at the leading edge of stalked barnacles, its presence also cannot be ruled out. However these proteins arrive in the adhesive, a critical issue is whether they perform a function that contributes to either adhesion or to some other activity at the interfacial region. It is noteworthy that some enzymes, specifically oxidases, do appear to maintain their function long after the adhesive is formed in *A. amphitrite* [21], lending support to the idea that proteins present in the adhesive can actively perform their biochemical function.

Many of the identified proteins can be associated with the immune system. Arthropods possess an innate immune system that functions by finding and destroying pathogens through a variety of means, including lysis, agglutination, melanization and phagocytosis [77]. The phenoloxidase cascade can be activated by multiple prophenoloxidase activating factors, including serine proteases [78] and apolipophorins [67]. Hemocytin [66], vitellogenin [68], apolipophorin [79], single domain von Willebrand factor type C [69] and C-type lectin [71] proteins bind to carbohydrates found on pathogen cells to initiate agglutination. Proteins involved with immunity and wound healing also overlap with cuticle sclerotization and have been associated with barnacle metamorphosis [80], but may have evolved novel adhesive roles in barnacles [20]. It is certainly possible that these proteins serve multiple functions including the promotion of protein agglutination as the adhesive interface expands.

The α-macroglobulin type pheromones, MULTIFUNCin and SIPC, may also have evolved from ancestral immune functioning α-macroglobulins [58]. α_2_-Macroglobulins clear circulating proteases [62] and contain a set of specific conserved domains which are present in various combinations in the pheromones identified in the adhesive of *P. pollicipes* and *A. amphitrite*. The extent of this modularity is greater in the pheromones identified from the *P. pollicipes* adhesive samples. Regions of AaSIPC have been shown to be important for promoting and dissuading cyprid settlement [81] as removal of 600 amino acids from the N-terminus, which corresponds to deletion or disruption of the A2M_N, MG3 and A2M_N_2 domains, induced avoidance behaviour. Previous research has also established that α-macroglobulin pheromones act as single proteins [59] or complexes [58]. The potential number of α-macroglobulin pheromones with conserved domain modularity combined with the possibility that multiple smaller proteins could form complexes and the added complexity of post-translation modification via glycosylation all point to a highly complex and adaptable protein-based pheromone communication that differs between stalked and acorn barnacles.

Beyond proteins that either function in immunity or sclerotization, several identified proteins suggest the potential for ligand binding or involvement in adhesion. Proteins with extracellular acetylcholine receptor (AChR), fasciclin and annexin domains were identified in both species, as were several predicted proteoglycans. These proteins and many others can be involved in extracellular matrix (ECM) formation or remodelling, suggesting its presence in the adhesive samples. The make-up of ECM includes large glycoproteins that polymerize to provide structure to the matrix, so it is noteworthy that two of the *P. pollicipes* proteoglycans are annotated as basement membrane-specific heparan sulfate proteoglycans. Formation and remodelling of the ECM is controlled by a diverse set of proteins, including some identified in the present study that include AChR containing proteins [82], fasciclin via integrin binding [72] and proteases that degrade ECM connective tissue [83]. A cyclophilin identified here was also noted in the proteomic analysis of a crayfish chitinous ECM [84]. Since the cuticle is an extracellular matrix produced by a layer of epithelial cells [85], the proteins described may be important regulators in this region.

The chitinous ECM also likely plays an important role in barnacle adhesion as adhesive proteins not only bind to the substrate but either directly or indirectly interact with the cuticle. As acorn barnacles grow, cells at the leading edge release a number of biomacromolecules (including protein) that are deposited at the leading edge of the barnacle base and under the developing cuticle [17]. The folded cuticle, created during a previous moult cycle, stretches flat and is observed pulling the biomacromolecules across the substrate. The old cuticle eventually tears, revealing a newly formed cuticle ready for expansion. Stalked barnacles also have a thick cuticle lining the base of the stalk [33]. The entire process of moulting, from the formation of a new cuticle to the breakdown of the old cuticle, is a dynamic process requiring a number of enzymes and structural proteins. Proteins with chitin-binding domains are integral components of the cuticle [86] and were identified in the adhesives of both *A. amphitrite* and *P. pollicipes*. Furthermore, oxidation plays an important role during arthropod cuticular sclerotization [87], which supports the identification of multiple oxidases in this study. Multiple types of enzymes are involved in cuticular degradation, including chitinases and serine proteases [88]. Proteomic analysis of the moulting fluid from the silk moth *Bombyx mori* revealed that chitinases, serine proteases, peptidases and protease inhibitors are important regulators of moulting [89]. Here, all of these enzymes were identified in the adhesive of both barnacle species with the exception of chitinases, which were only identified in *P. pollicipes*. These results provide further support for the idea that the adhesive material of *A. amphitrite* and *P. pollicipes* collected for proteomics analysis also contains cuticular material and suggests that the interaction between the cuticle and the substrate interface proteins is critical for the proper function of adult barnacle permanent adhesive and, ultimately, survival of the animal. This tracks with the observations of the cyprid adhesive in acorn barnacles [90].

The non-homologous bulk proteins have garnered the most scientific interest as they are thought to function as the actual adhesive at the interface [8,23,51]. While an in-depth discussion of how each type of bulk protein (note: we are grouping together proteins previously termed bulk and interfacial proteins for simplicity [14]) could contribute to adhesion is outside the scope of this work, this topic has been discussed elsewhere [7,8,21,33]. In the current work, more unique bulk proteins were identified in the adhesive proteome of *A. amphitrite* without homologues identified in the *P. pollicipes* genome. Also, several of the *A. amphitrite* bulk protein families contain more individually identified members. Stalked barnacles are closer to the ancestral node than acorn barnacles on the barnacle tree of life [29], so these differences may be evidence of an expansion of these specialized proteins in acorn barnacles. Despite this, the amino acid composition and the potential for glycosylation of the glycine and leucine-rich bulk proteins show no clear differences between the species, suggesting that certain properties of bulk proteins have been conserved between acorn and stalked barnacles. Additionally, approximately 70% of the identified bulk proteins do have a homologous partner in the adhesive proteome of the other species, again supporting the extent of conservation between these adhesive proteomes.

Using recently assembled genomes to re-examine the adhesive proteome of representative stalked and acorn barnacles, this work represents a new level of analytical detail into the make-up and comparison of barnacle adhesive. Our work highlights the overall similarity in the types of proteins present in the adhesive of these distantly related barnacles while revealing that the largest differences between the species exist in the specialized bulk proteins. These observations are especially striking given that these similarities are being observed between a basal (*P. pollicipes*) and a highly derived (*A. amphitrite*) barnacle species. As omic sequencing continues to improve, both in the types of species that are sequenced and the quality of the sequencing data, this work provides a roadmap to reveal the fundamental biomolecular contributors to one of nature's most robust marine adhesives. Quantification of the amino acid composition of the entire adhesive for each species [91] and the abundance of each type of protein, as well as *in vitro* experiments examining species specific differences in fibril formation [18,19] or other traits are additional steps that will significantly contribute to understanding the role of bulk proteins in adhesion and whether these proteins have become more specialized across barnacle lineages. Finally, continued improvements in sample preparation, for both gDNA/mRNA and proteins, and the application of standard collection and sample processing methods to the adhesive from different species will enhance the ability for direct comparison of different species, highlighting both the similarities and interspecies differences in the adhesive proteome.

## Data accessibility

All raw mass spectrometry files associated with this study are available externally at the ProteomeXchange Consortium (*Amphibalanus amphitrite*: Sciex files: PXD012730; Oribtrap files: PXD026105) or Mendeley Data (*Pollicipes pollicipes*: http://dx.doi.org/10.17632/pgkf3mtb4m.1). All results files from MaxQuant are included as electronic supplementary material, files S2–S6.

The data are provided in electronic supplementary material [92].
